# Minor Splicing Factors *Zrsr1* and *Zrsr2* Are Essential for Early Embryo Development and 2-Cell-Like Conversion

**DOI:** 10.3390/ijms21114115

**Published:** 2020-06-09

**Authors:** Isabel Gómez-Redondo, Priscila Ramos-Ibeas, Eva Pericuesta, Raúl Fernández-González, Ricardo Laguna-Barraza, Alfonso Gutiérrez-Adán

**Affiliations:** Departamento de Reproducción Animal, INIA, Avda. Puerta de Hierro n° 12. Local 10, 28040 Madrid, Spain; igomer00@gmail.com (I.G.-R.); priscilaramosibeas@gmail.com (P.R.-I.); pcamacho@inia.es (E.P.); raulfg@inia.es (R.F.-G.); rilaba@hotmail.com (R.L.-B.)

**Keywords:** *Zrsr1*, *Zrsr2*, U12 introns, zygotic gene activation, 2C-like cells

## Abstract

Minor splicing plays an important role in vertebrate development. *Zrsr1* and *Zrsr2* paralog genes have essential roles in alternative splicing, mainly participating in the recognition of minor (U12) introns. To further explore their roles during early embryo development, we produced *Zrsr1^mu^* and *Zrsr2*^mu^ mutant mice, containing truncating mutations within the second zinc finger domain. Both homozygous mutant mice were viable with a normal lifespan. When we crossed a homozygous *Zrsr2*^mu/mu^ female with *Zrsr1*^mu/mu^ male, the double heterozygotes were non-viable, giving rise to embryos that stopped developing mainly between the 2- and 4-cell stages, just after zygotic gene activation. RNA-seq analysis of *Zrsr1/2^mu^* 2-cell embryos showed altered gene and isoform expression of thousands of genes enriched in gene ontology terms and biological pathways related to ribosome, RNA transport, spliceosome, and essential zygotic gene activation steps. Alternative splicing was analyzed, showing a significant increase in intron retention in both U2 and U12 intron-containing genes related to cell cycle and mitotic nuclear division. Remarkably, both *Zrsr1* and *Zrsr2* were required for the conversion of mouse-induced pluripotent stem cells into 2C-like cells. According to our results, *Zrsr1* or *Zrsr2* are necessary for ZGA and both are indispensable for the conversion of induced pluripotent stem cells into 2C-like cells.

## 1. Introduction

Alternative splicing (AS) is an important co- and post-transcriptional process through which multiple transcripts are generated from a single gene. There are two splicing machineries: the major class or U2-dependent spliceosome, which removes the majority of introns (U2-type intron); and the minor class or U12-dependent spliceosome, which removes U12-type introns (<0.4% of all introns) [1]. U12-type introns are non-randomly distributed across the genome, and despite their scarce abundance, are highly conserved across distantly related eukaryotic taxa, indicating their common evolutionary origin [2,3]. Further, genes with U12 introns are over-represented in functions and pathways related to development, such as RNA processing, DNA replication, or cell cycle [4,5]. Mutations within both the protein and snRNA components of the minor spliceosome have been associated with multiple diseases, including developmental disorders [4,6,7,8,9], neurodegeneration [10], and cancer [11]. Additionally, the minor spliceosome is thought to play an important developmental role in plants [12,13,14], drosophila [15] zebrafish [16], and in the mouse central nervous system [17,18], hypothalamus [19], gametogenesis [9], and early development [20]. Systematic transcriptome and proteome analyses of mouse and human preimplantation embryos have revealed that genes involved in mRNA splicing are over-represented during early preimplantation development [21], before and during zygotic gene activation (ZGA) [22]. However, the specific role of minor splicing in preimplantation embryo development has not been fully elucidated.

The splicing factors ZRSR1 (also known as U2af1-rs1) and ZRSR2 (also known as U2af1-rs2) (encoded by the *Zrsr1* and *Zrsr2* genes) have been attributed essential roles in splice site recognition of U12 and U2 introns [23]. These paralogs have been identified in all analyzed mammalian species [9,24] and are known to recognize the 3’AC dinucleotide of the AT-AC class of U12-type introns or the 3’AG of U2-type introns [23]. Recent sequencing studies have identified frequent somatic *ZRSR2* mutations in hematological malignancies, such as myelodysplastic syndrome (MDS), causing mis-splicing of U12 introns [11]. However, their precise role in embryo development and function in other pluripotent cell types remains unclear. *Zrsr1* is an intronless gene located on mouse chromosome 11 and on chromosome 5 in humans, in whom it is considered a pseudogene (*ZRSR2P1*). Murine *Zrsr1* is a retrotransposed copy of X-linked *Zrsr2* (located on the X chromosome in all mammalian species analyzed) and is paternally expressed in the placenta and some adult tissues, while the maternal copy is methylated and silent. *Zrsr1* knockout mice show no abnormal phenotype [25], probably because of the expression of the other paralog. However, we recently observed that *Zrsr1* plays key roles in hematopoiesis, as *Zrsr1^mu^* mice showed severe defects in red blood cells [9]. Muscle strength was also affected and alterations at the spermatocyte stage were observed, leading to infertility in almost 95% of males, affecting U12 introns [9] and revealing functional similarities between *Zrsr1* and *Zrsr2*. 

To gain further insight into the role of *Zrsr1* and *Zrsr2* in preimplantation development, in the present study we generated *Zrsr1* and *Zrsr2* mutant mice, containing truncating mutations in their RNA recognition motif. Double mutants produced by crossing males homozygous for the *Zrsr1* mutation with females homozygous for the *Zrsr2* mutation stopped developing just after ZGA, and showed enhanced intron retention affecting U2 and U12 introns and exon skipping. Rescue experiments in which *Zrsr1* mRNA was injected into single-cell double mutant embryos extended the development of mutant embryos, revealing that minor splicing is essential for ZGA. Collectively, this study identifies ZRSR1 and ZRSR2 as essential factors for efficient U2 and U12 intron splicing and reveals their crucial roles in genome activation during both ZGA and conversion of induced pluripotent stem cells (iPSC) into 2C-like cells (2CLC). 

## 2. Results

### 2.1. Double Zrsr1/Zrsr2 Mutant Embryos Are Non-Viable as Maternal Zrsr2 or Paternal Zrsr1 Are Necessary for Early Preimplantation Development

To examine the implications of minor splicing during preimplantation development, we first addressed the expression of U2- and U12-type introns during mouse preimplantation development using published RNA-seq data [26]. While ~35% of genes were expressed before implantation, more than 60% of genes carrying a U12 intron were expressed at all stages of preimplantation development (Supplementary Figure S1a). RT-qPCR showed that *Zrsr1* expression was higher at the 2-cell stage (at the time of ZGA) and at the blastocyst stage [9]. In contrast, *Zrsr2* was present in the oocyte but showed lower expression during preimplantation embryo development (Supplementary Figure S1b). Similar expression profiles were observed in human and bovine embryos (Supplementary Figure S1c,d; data from [27] and [24,28], respectively). These expression profiles point to an active role of the minor spliceosome in early development in mammals, mainly during ZGA and at the blastocyst stage.

We used CRISPR-Cas9 technology to produce *Zrsr2* mutant mice with a nonsense mutation in the second zinc finger motif (ZnF2), which is required for splicing regulation and control of protein stability and contributes to the interaction with U2af2 [29]. We obtained three transgenic lines with different deletions that produced differently sized mutant proteins that were viable and fertile in homozygosis, showing no phenotypic differences. For this study, we used the transgenic line 3 *Zrsr2^mu3^* (hereafter designated *Zrsr2^mu^*), which has a 25 nucleotide deletion in exon 10, losing ZnF2 and the final region of the protein. *Zrsr1^mu^* mice were generated as described by Horiuchi et al. (2018) [9].

Interestingly, although the homozygous mutation of *Zrsr1* or *Zrsr2* does not affect lifespan (Supplementary Figure S2a) (*p* > 0.5, Student’s *t*-test), when we crossed double heterozygous *Zrsr1*^WT/mu^/*Zrsr2*^WT/mu^ females with double heterozygous *Zrsr1*^WT/mu^/*Zrsr2*^mu/y^ males (10 females crossed with 5 males), no double homozygotes with both mutations were born (*n* = 73 animals analyzed). To explore the mechanisms involved in embryo losses, we crossed *Zrsr2*^WT/mu^ heterozygous females (Supplementary Figure S2b) or double *Zrsr2*^WT/mu^ *Zrsr1*^WT/mu^ heterozygous females (Supplementary Figure S2c) with fertile *Zrsr1*^mu/mu^ males (28 females crossed with 14 males) and genotyped the pups (*n* = 69 for b and *n* = 75 for c). No animals with the mutant *Zrsr2* allele were born. It should be noted that because *Zrsr1* is maternally imprinted in mice, embryos can only express the mutant *Zrsr1* from the paternal allele and *Zrsr2* from 2 (WT or mutant in females) or 1 (WT in males) alleles (present in the X chromosome). Thus, when the paternal *Zrsr1* mutant allele is expressed in the embryo, the expression of one *Zrsr2* WT allele from the mother is necessary for its survival (Supplementary Figure S2b,c). To address these issues, we crossed homozygous *Zrsr2*^mu/mu^ females (*n* = 15; fertile when crossed with a WT or a *Zrsr2^mu/y^* male) with fertile *Zrsr1*^mu/mu^ males. No offspring was born, confirming that when the embryo expresses the *Zrsr1* mutant allele from the father it needs one WT *Zrsr2* allele from the mother (Supplementary Figure S2d). This is consistent with a report indicating that the *Zrsr2* allele obtained from the father is inactive during early preimplantation development [30]. These results suggest that *Zrsr1* and *Zrsr2* are complementary during early development and that embryos need just one WT *Zrsr1* allele from the father or one WT *Zrsr2* from the mother to survive.

To determine the moment of embryo loss, we examined embryos obtained from *Zrsr2*^mu/mu^ females crossed with *Zrsr1*^mu/mu^ males. Preimplantation development was arrested in all the double mutant embryos (hereafter designated *Zrsr1/2*^mu^), mainly at the 2-cell stage (during ZGA), while embryos developed normally in the opposite mating. Only 20% (51 of 258) developed into morulae, and when transferred to surrogate mothers these morulae did not implant (285 embryos analyzed from 10 different matings). However, when in-vitro-produced *Zrsr1* mRNA was microinjected into the cytoplasm of *Zrsr1/2*^mu^ zygotes, more than 60% (121 of 197 injected vs. 5 of 129 non-injected; *p* < 0.01, z-test) of the embryos developed to the morula and blastocyst stages, although after embryo transfer they were unable to develop. These results indicate that extra *Zrsr1* WT could offset the loss of ZRSR2 function during 2-cell ZGA, rescuing preimplantation development.

### 2.2. Transcriptome Analysis of Double Zrsr1/2 Mutant Embryos at the 2-Cell Stage

**Figure 1:**
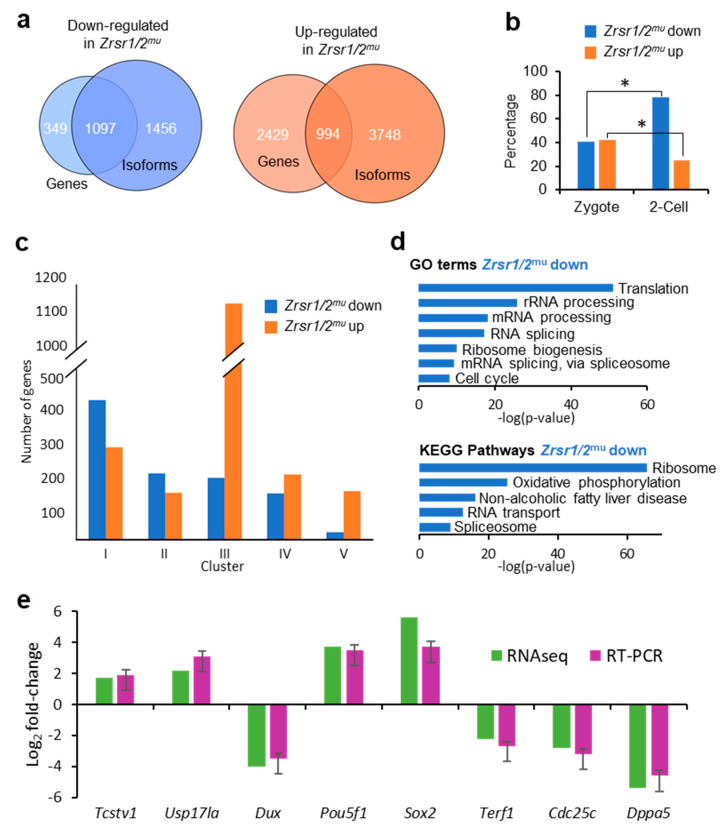
RNA-seq analysis of *Zrsr1/2*^mu^ embryos, gene ontology, and RT-PCR validation. (**a**) Venn diagrams comparing up- (orange) or downregulated (blue) genes and isoforms in *Zrsr1/2*^mu^ vs. wild-type (WT) 2-cell embryos. (**b**) Percentage of genes up- or downregulated in *Zrsr1/2*^mu^ that are present in zygote or 2-cell stages in mice [30]; *p < 0.01 (z test). (**c**) Clustering of differentially expressed genes according to [31]. (**d**) Significant gene ontology (GO) terms (upper panel) and Kyoto Encyclopedia of Genes and Genomes (KEGG) pathways (lower panel) in differentially expressed genes (DEGs) in the pair-wise comparison are indicated. (**e**) Validation of RNA-seq data for differential gene expression by qRT-PCR of selected RNAs from *Zrsr1/2^mu^* and WT 2-cell embryos (3 pools of 10 2-cell embryos; triplicate results are presented as the mean ± SEM). No statistically significant differences were found by ANOVA followed by Tukey’s post hoc test (*p* < 0.05).

To determine the molecular basis of the developmental arrest observed in *Zrsr1/2*^mu^ embryos, we undertook RNA-seq analysis of WT and *Zrsr1/2^mu^* embryos. Around 24,000 genes and 50,000 transcripts were detected per sample, while 60% of U12-containing genes were expressed. In *Zrsr1/2*^mu^ embryos, 3423 and 1446 genes were up- and downregulated, respectively (Supplementary Tables S1A and S2, Figure S1a). The large number of upregulated genes suggests that maternal mRNAs were not efficiently degraded in the mutant embryos. When we examined differentially expressed isoforms (DEIs), showing that 6970 and 4491 isoforms were up- and downregulated, respectively (Supplementary Tables S1B and S3, Figure S1a). Comparisons between differentially expressed genes (DEGs) and coding DEIs revealed that ~30% and ~76% of up- and downregulated genes also showed significant changes in isoform expression between groups (994 and 1097, respectively; Supplementary Table S1C, Figure 1a). Eighty-six out of 994 and 84 out of 1097 only had one isoform. When genes upregulated in wild-type (WT) or *Zrsr1/2*^mu^ embryos were compared with previous data for genes expressed exclusively in zygotes or 2-cell embryos [30] (Figure 1b), the percentage of genes upregulated in *Zrsr1/2*^mu^ over genes normally expressed in 2-cell embryos was significantly lower than that of genes expressed at the zygote stage, while more genes upregulated in WT were in common with those reported at the 2-cell embryo stage than zygote stage, as expected. Thus, the larger number of genes and isoforms upregulated in *Zrsr1/2*^mu^ embryos could be due to blockage in the degradation of the oocyte’s maternal mRNA or to alterations in ZGA and progression to the 2-cell stage. We also compared our data with the dynamics of gene expression during ZGA according to the clustering described in [31] (Figure 1c), obtaining a remarkably high number (1138) of genes upregulated in *Zrsr1/2*^mu^ embryos that correspond to cluster III, which includes genes whose expression levels decrease continuously from fertilization and to the 2-cell stage. Taken together, these results support the idea that maternal mRNAs are not being fully degraded.

Genes that showed reduced expression in *Zrsr1/2*^mu^ were mainly enriched in gene ontology (GO) terms related to translation, mRNA processing, and splicing; and in Kyoto Encyclopedia of Genes and Genomes (KEGG) pathways related to the ribosome, RNA transport, spliceosome, and essential ZGA steps [21] (Figure 1d and Supplementary Table S9). However, genes that showed increased expression in *Zrsr1/2*^mu^ did not feature significant enrichment in GO terms or KEGG pathways. We validated the RNA-seq results by examining the expression of 4 downregulated genes and 4 upregulated genes. All genes showed similar expression between RNA-seq and RT-qPCR (Figure 1e).

### 2.3. U12 Intron Retention Is the Most Frequent Splicing Event Observed in Double Mutant Zrsr1/2mu 2-Cell Embryos

**Figure 2:**
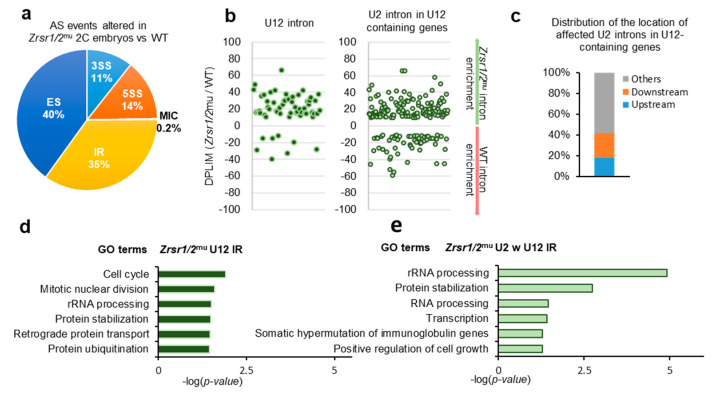
RNA-seq analysis of alternative splicing (AS) events in *Zrsr1/2*^mu^ 2-cell (2C) embryos. (**a**) Distributions of categories of AS events differing in *Zrsr1/2*^mu^ embryos versus WT. The percentage of each class of events is indicated. Note: 3SS, alternative 3′ splice sites; 5SS, alternative 5′ splice sites; ES, exon skipping; MIC, alternative micro cassette exon ≤15 nucleotides; IR, intron retention. (**b**) Differences in intron retention events detected in *Zrsr1/2*^mu^ compared to WT embryos (measured as the ratio of intron read counts (DPLIM) in *Zrsr1/2*^mu^ versus WT) for different intron categories, as indicated. (**c**) Distributions of the locations of affected U2 introns in *Zrsr1/2*^mu^ embryos relative to U12 introns present in the same gene. (**d**) Significant GO terms for U12-type introns represented in graph “B” to the left. (**e**) Significant GO terms for U2 introns within (w) U12-containing genes represented in graph “B” to the right.

Alternative splicing events were categorized into 5 different groups: 3′ AS site (3SS), 5′ AS site (5SS), exon skipping (ES), intron retention (IR), and micro-exon skipping or alternative microcassette exon ≤15 nucleotides (MIC) based on their inclusion levels. Overall, 4362 differentially spliced events were identified, 2645 upregulated and 1717 downregulated in *Zrsr1/2*^mu^ embryos (Supplementary Table S1D and S5). All categories of alternative splicing events were affected (Figure 2a). Similar numbers of U2 IR events were found up- and downregulated in *Zrsr1/2*^mu^ embryos (Supplementary Table S1D). Fifty-three out of 710 IR events upregulated in *Zrsr1/2*^mu^ embryos corresponded to the U12 class of intron, a large enrichment over the overall proportion of U12 introns in the mice (~7% vs. ~0.04% expected, Figure 2b). Sixteen percent (114 out of 710 events) of the IR upregulated in *Zrsr1/2*^mu^ embryos corresponded to U2 introns within U12-containing genes, with ~40% of them being located immediately upstream or downstream from the U12 introns (Figure 2c), also suggesting an interplay between major and minor spliceosomes. Interestingly, all the alternative events modified in *Zrsr1/2*^mu^ embryos appeared to be enriched in U12 intron-containing genes, except MIC and 5SS (Supplementary Figure S2e). U12 IR events observed in *Zrsr1/2*^mu^ embryos were confirmed by quantitative RT-PCR, where 11- to 52-fold increased expression was detected in mutants compared with WT for 6 out of 8 U12 introns examined (Supplementary Figure S2f). We observed no changes in the retention of *Gpaa1* and *Hdac10* U2 introns.

GO terms of genes that showed U12 intron retention in *Zrsr1/2*^mu^ (Figure 2d) and genes containing U2 introns within U12 (Figure 2e) indicated that the defect in ZGA could be, at least in part, attributed to the misregulation of cell cycle and mitotic genes containing U12 introns. Candidate genes containing affected U12 introns that could underlie this cleavage blocking include components of the RNA polymerase complex (*Polr1e*, *Polr2e*, and *Polr3c*), components of the DNA polymerase complex and DNA replication (*Pole2, Prim1, Nol8*), genes involved in the base excision repair (BER) pathway (*Parp1*), in mismatch repair and recombination (*Exo1*, *Msh3*), and in the translation preinitiation complex (*Eif3k*, *Mcts1*), among others.

### 2.4. Zrsr1 and Zrsr2 Are Necessary for the Conversion of Induced Pluripotent Stem Cells (iPSCs) to 2C-Like Cells

**Figure 3:**
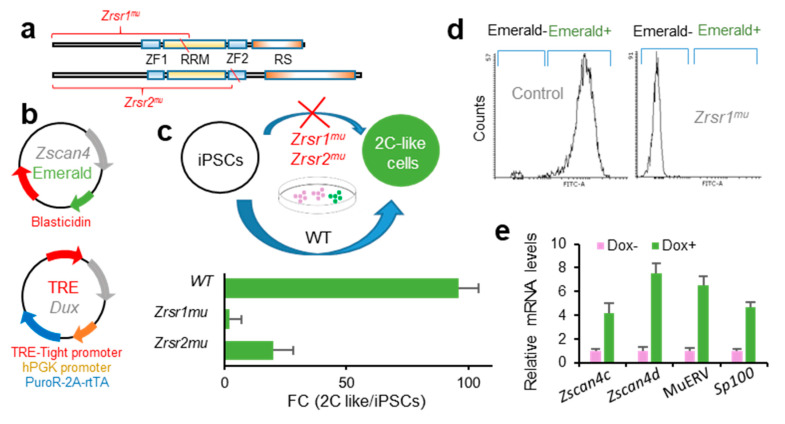
*Zrsr1*^mu^ and *Zrsr2*^mu^ block activation of the 2-cell transcription program induced by Dux expression and impair conversion of induced pluripotent stem cells (iPSC) to a 2 cell-like cells (2CLC) state. (**a**) Diagram of *Zrsr1* and *Zrsr2* mutants with their functional domains: ZF: Zinc Finger, RRM: RNA recognition motif, RS: arginine-serine rich. Diagonal red line indicates the position of the mutation. (**b**) Diagram of the plasmid-expressing Emerald under *Zscan4* promoter [37] and of a doxycycline-inducible lentiviral construct [27]; both plasmids were stably integrated into iPSC. (**c**,**d**) Diagram of iPSC metastability (top) and enrichment in 2CLCs relative to conventional iPSCs (bottom) produced after Dux expression in control iPSCs but blocked when *Zrsr1* or *Zrsr2* are mutated (**c**), as confirmed by flow cytometry (**d**) using two cell cultures per condition. (**e**) Relative mRNA expression in WT Dux-induced iPSCs (2CLCs) of genes related to 2-cell embryo genome activation.

*Zrsr1* and *Zrsr2* are expressed in iPSCs [32], where *Zrsr1* methylation has been related to pluripotency [33]. Pluripotent cells resembling 2-cell-stage embryos (2C-like cells, 2CLC) are a rare metastable cell population that appears at very low frequency in mouse embryonic stem cells (mESCs) and iPSCs in culture. These totipotent cells express markers of 2-cell stage embryos, such as *Zscan4* family genes, and have been identified and isolated based on their spontaneous reactivation of murine endogenous retrovirus with leucine tRNA primer binding site (MERVL), a mouse-specific retrotransposon otherwise expressed only in 2-cell embryos [34,35]. The conversion of mESCs to the 2CLC state is driven by Dux by facilitating chromatin accessibility at MERVL elements, which are used specifically in 2-cell embryos to regulate gene expression during ZGA [27]. Here, we used a doxycycline-inducible Dux construction [27] to test whether *Zrsr1* and *Zrsr2* were necessary for the induction of 2CLC (Figure 3), as Dux expression was not affected in our 2-cell embryos RNA-seq analysis. We generated homozygous *Zrsr1*^mu^ and *Zrsr2*^mu^ mice (*Zrsr2*^mu3^ and *Zrsr2*^mu9^ lines that allow normal embryo development and offspring production in homozygosis) (Figure 3a) with a transient transcription factor (OSKM) transgene to produce iPSCs (i4F-B reprogrammable mouse line [36]). *Zrsr1*^mu^ and *Zrsr2*^mu^ iPSCs were then stably transformed with a construct expressing Emerald under the *Zscan4* promoter [37], the selected and again transformed with the dox-inducible Dux construct [27] (Figure 3b). Using two clone cell lines for each experimental group in two separate experiments, we quantified by flow cytometry the number of Emerald-positive cells 24 h after dox induction. Conversion efficiencies were significantly lower in *Zrsr1*^mu^ (2%) and *Zrsr2*^mu^ (20%) than in WT (96%) (Figure 3c,d). The 2CLC identity was confirmed in WT Dux-induced iPSCs through the expression of *Zscan4c*, *Zscan4d*, MERVL, and *Sp100*, which are expressed in 2-cell embryos and 2CLC mESC but show low expression in conventional mESCs or iPSCs (Figure 3e). The ZSCAN4 protein is expressed during chromatin reorganization in late meiotic prophase oocytes and spermatocytes [38], in the same spatiotemporal setting as when *Zrsr1* mutants block gametogenesis [9]. Interestingly, heterozygous *Zrsr1* or *Zrsr2* mutant lines showed normal 2CLC conversion efficiencies, indicating that the mutant form of the proteins is not acting as a dominant negative disruptor and that the observed effects are due to the loss of function of the mutant proteins. Taken together, our data indicate that *Zrsr1* and *Zrsr2* are necessary for the generation of 2CLC, and contrary to what happens with the embryos, *Zrsr1* and *Zrsr2* are not complementary in iPSCs, as both are required for the induction of 2CLC.

## 3. Discussion

Minor splicing is known to have essential roles in vertebrate development. Through a systematic analysis of the transcriptome of mouse embryos with mutations in ZRSR1 and ZRSR2 minor splicing factors, we have discovered that there is a clear complementary action between *Zrsr1* and *Zrsr2* during 2-cell embryo cleavage. However, this does not happen during 2CLC reprogramming from iPSCs, where mutations in just one of the genes block their conversion. This could be explained by the mutation in one of the genes affecting the functionality of the other, or by differentiation of their expression or functions in this process. Interestingly, although *Zrsr1^mu^* and *Zrsr2^mu^* iPSCs could not be reprogrammed to the 2CLC stage, they could be derived and expanded for more than 30 passages without apparent loss of viability.

According to our RNA-seq results, there is a remarkably high number of overexpressed genes in the *Zrsr1/2^mu^* embryos that are supposed to be degraded during early embryo development [31,39], strongly suggesting that there is a blockade in the degradation of the oocyte’s maternal mRNAs. Moreover, our GO analysis of U12 intron-bearing genes with alterations in intron retention in *Zrsr1/2^mu^* embryos revealed strong enrichment of genes related to key cellular functions, highlighting the importance of these genes in the 2-cell blockade. *Zrsr1/2^mu^* also indirectly modified the expression or produced IR of many other genes carrying U12 introns reported to play important roles in embryo cleavage, DNA damage, and conversion of iPSCs to 2CLC (e.g., *Esrp1*, *Srsf10*, *Cul1*, etc.).

According to a recent study, minor splicing is essential for preimplantation development [20]. Knockout of *Rnpc3*, coding for one of seven proteins unique to the U12-dependent spliceosome carrying a U12 intron, blocks preimplantation development in mice [20]. These authors indicate that embryos fail to develop beyond the morula stage, similar to our results obtained in *Zrsr1/2^mu^* mice, where only a few embryos continue developing via abnormal divisions after the 2-cell stage until the morula stage. In the study by Doggett et al. [20], the mother was heterozygous for the *Rnpc3* mutation, and thus the embryo could undergo the first few divisions using the protein product of the WT maternal allele in the oocyte, reaching a further developmental stage than in our experiment. We also showed the complementary behavior of *Zrsr1* and *Zrsr2* during preimplantation development. To ensure their survival, embryos need at least one *Zrsr1* WT allele from the father (as it is maternally imprinted and only expressed from the paternal allele) or one *Zrsr2* WT allele from the mother (as only *Zrsr2* is expressed in the oocyte and the embryo needs the protein during early stages of development). Further, since *Zrsr2* is located on the X-chromosome, the *Zrsr2* allele passed on from the father is inactivated during early preimplantation development, because X chromosome inactivation (XCI) is imprinted during early preimplantation development, with the paternal X chromosome (Xp) being initially inactivated and then reactivated during the formation of the inner cell mass (ICM) at the blastocyst stage [40]. *Zrsr1* is an intronless gene in both humans and mice, and both species show high *Zrsr1* expression after ZGA, in agreement with the conserved trend of intron-poor transcripts being among the first genes that are highly expressed in the zygote [41]. Additionally, the genes and proteins involved in mRNA splicing are over-represented during early embryo cleavage [21] and are essential for conversion to 2CLC. Epigenetic regulators of 2CLC conversion have been recently identified [42]. The top 49 genes in this list include 23 components of the spliceosome, highlighting the importance of splicing in this process. Sixteen of these genes display abnormal AS or downregulated expression in *Zrsr1/2^mu^* embryos and 5 of them have U12 introns (*Ddx18*, *Ints4*, *Snrpb*, *Snrpe*, and *Ubl5*; *Ints4* with IR in 2 U12 introns and *Ubl5* with IR in one U2 intron).

**Figure 4:**
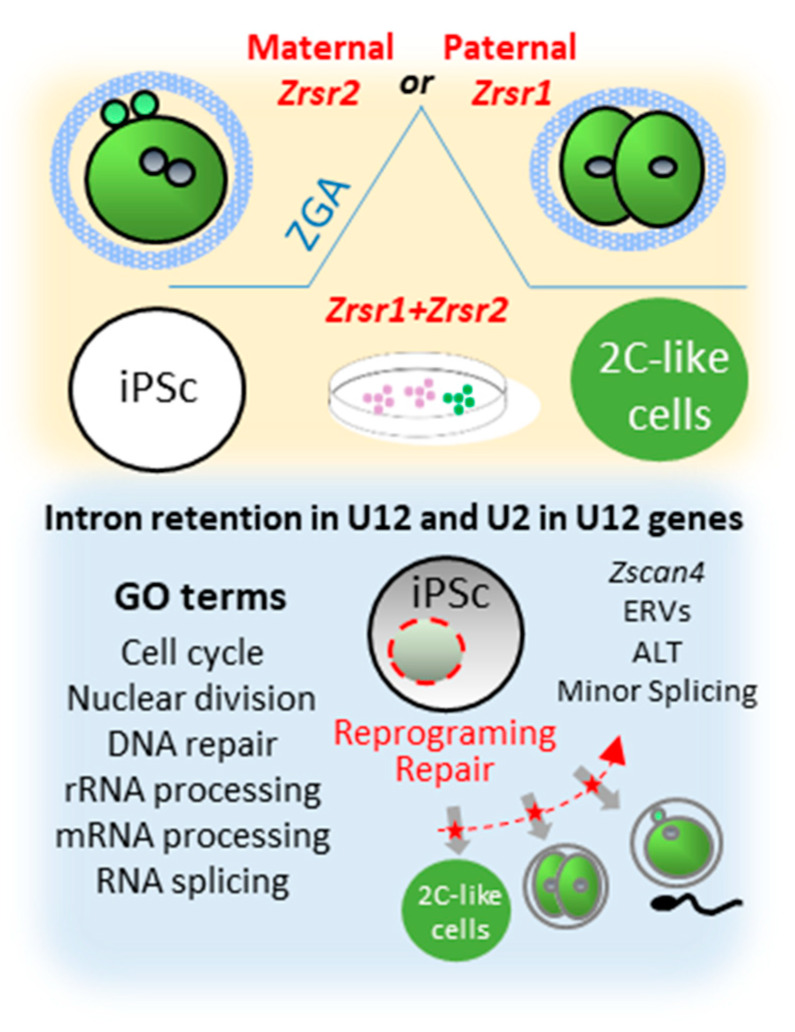
Synopsis of phenotypes produced by *Zrsr1* and *Zrsr2* mutations. Upper panel shows that mutation of maternal *Zrsr2* and paternal *Zrsr1* blocked early embryo development during zygotic gene activation (ZGA) and increased intron retention in U2- and U12-containing genes; and that mutation of either *Zrsr1* or *Zrsr2* led to a blockade of induced pluripotent stem cell (iPSC) conversion to 2C-like cells. The bottom panel shows gene ontology (GO) enrichment terms related to these phenotypes, indicating that disturbed U12-containing genes are critical for cell division, differentiation, and DNA damage response.

We have previously described the role of the minor splicing factor *Zrsr1* in spermatogenesis [9] and in the organization of the hypothalamic cell network controlling behavior [19], showing that altered minor splicing has a sex-dimorphic effect in social behavior. Nevertheless, the significance of minor splicing during preimplantation embryo development is still poorly understood. This comprehensive analysis of *Zrsr1/2* mutant mouse embryos reveals a new role of paternal *Zrsr1* and maternal *Zrsr2* as components of the ZGA mechanism in the embryo, and mutation of either *Zrsr1* or *Zrsr2* blocked the reprograming of iPSCs towards 2CLC (Figure 4). Accordingly, *Zrsr1* and *Zrsr2* emerge as essential for stem cell differentiation during gametogenesis in both males and females [9], totipotent zygote differentiation (ZGA), and reprogramming of iPSCs towards 2CLC, suggesting a critical role of minor splicing in the stem cell reprogramming process.

## 4. Materials and Methods

### 4.1. Animals

*Zrsr2^mu^* mice were generated by inducing a nonsense mutation in the second zinc finger motif (ZnF2) of the *Zrsr2* gene. Cas9-10DA mRNA and sgRNAs (Supplementary Table S7) were produced (Sigma-Aldrich, MI, USA) and injected into B6BAF1 (C57BL/6xCBA) zygotes, which were transferred to pseudo-pregnant females at the 2-cell or blastocyst stage [43]. Pups were genotyped by PCR in standard conditions with specific primers (Supplementary Table S7). *Zrsr1^mu^* mice were generated as described by Horiuchi et al. (2018) [9]. *Zrsr1/2^mu^* embryos were obtained by crossing double-heterozygous *Zrsr2*^mu/mu^ females with *Zrsr1*^mu/mu^ males. Animal experiments were performed in accordance with European Community Council Directive 2010/63/EU guidelines, sanctioned by the Committee on the Ethics of Animal Experiments of the INIA (Madrid), and licensed by the Animal Protection Area of the Counseling of Environment of the Community of Madrid (Spain), in accordance with Statutory Instrument ref PROEX 261/15 of July 2015. 

For the rescue experiments, mouse Zrsr1 subcloned into pcDNATM5/FRT/TO inducible expression vector (ThermoFisher Scientific, Waltham, MA, USA) [9] was transcribed with mMESSAGE mMACHINE T7 Ultra Kit (ThermoFisher Scientific, Waltham, MA, USA). RNA was purified using MEGAclear Kit (ThermoFisher Scientific, Waltham, MA, USA) and frozen at −80 °C. B6CBAF1 zygotes were injected using a Piezo impact-driven micromanipulator (Prime Tech Ltd., Ibaraki, Japan). After microinjection, zygotes were cultured in potassium simplex optimized medium (KSOM, Sigma) in a humidified atmosphere of 5% CO_2_ and 95% air at 37 °C. Blastocysts were transferred to CD1 recipients by in utero transfer [44].

### 4.2. RNA Extraction and RNA-Seq Analysis of Embryos

Total RNA was extracted from 3 pools of ~100 *Zrsr1/2*^mu^ 2-cell embryos and 2 pools of ~100 wild-type 2-cell embryos (produced in different experimental repetitions) using Arcturus Pico Pure RNA Isolation Kit (Molecular Devices, San Jose, CA, USA). The purified total RNA was stored in nuclease-free water and then used for the first-strand synthesis. RNA concentration was measured using a Qubit^®^ RNA Assay Kit in a Qubit^®^ 2.0 Fluorimeter (ThermoFisher Scientific, Waltham, MA, USA). First-strand cDNA (from total RNA) was synthesized according to the SMART-Seq™ v4 Ultra™ Low Input RNA Kit protocol. The PCR-amplified cDNA was purified using AMPure XP beads, then 1 μL cDNA was validated using an Agilent 2100 Bioanalyzer. The cDNA samples were sheared with a Covaris system (Covaris, Woburn, MA, USA) before library preparation. Sequencing libraries were generated using a NEBNext^®^ Ultra™ DNA Library Prep Kit for Illumina^®^ (NEB, Ipswich, MA, USA) according to the manufacturer’s recommendations. In short, the workflow included the conversion of sheared DNA into blunt ends, adenylation of the DNA fragments’ 3′ ends, ligation of index-coded adapters, and PCR amplification. Finally, PCR products were purified (AMPure XP system) and library quality was assessed in an Agilent Bioanalyzer 2100 system (Agilent, Santa Clara, CA, USA) (effective concentration of the cDNA libraries >2 nM). These libraries were sequenced using an Illumina HiSeq platform with 150 bp paired-end sequencing at the Novogene Bioinformatics Institute (Beijing, China). The RNA-seq data generated are available via ArrayExpress (Accession number E-MTAB-9102). Raw data (raw reads) in fastq format were first processed with in-house Perl scripts. In this step, clean data (clean reads) were obtained by removal of reads containing adapter sequences, reads containing poly-N, and low-quality reads (Qscore ≤ 5) from the raw data. All the downstream analyses were based on clean, high-quality data. Next, each sample was aligned against the mouse reference genome and transcriptome (Mmu10/GrCm38) with STAR software v2.5 [45] and sorted with Samtools software [46]. On average, ~85 million stranded 150-bp paired-end sequencing reads of each sample were aligned.

#### 4.2.1. Differential Gene Expression Analysis

Pure read counts were calculated from the alignment files using HTSeq-count software v0.80 [47]. Differential gene expression analyses were performed independently using the R packages edgeR v3.22.3 [48] and DESeq2 v1.20.0 [49]. To improve the accuracy of our results, only genes identified by both programs with an adjusted *p*-value below 0.01 were considered as differentially expressed. Gene ontology enrichment analysis was performed with the David Gene Functional Classification Tool [50].

#### 4.2.2. Differential Isoform Expression Analysis

Raw transcript counts were calculated from our transcriptome alignment files using RSEM software v1.3.1 [51], with a forward probability of 0.5. Differential isoform usage was then analyzed using the Bioconductor package EBSeq, which addresses isoform expression estimation uncertainty by correcting for the differential variability present in distinct isoform groups [52]. Isoforms were considered differentially expressed when their FDR was below 0.01.

#### 4.2.3. Differential Splicing Analysis

To identify differentially spliced events in the two groups, levels of inclusion of each transcript in the mRNA were determined using vast tools [53], normalizing the distribution of each AS event to the overall number of that event in the mouse transcriptome (mm10 annotation). AS events differentially spliced in the two groups were then identified by calculating the differences in their average inclusion levels (ΔPSI), removing those events with low read coverage. Events whose ΔPSIs were higher than 10% were considered differentially spliced and classified as exon skipping (ES), alternative 3′splice site (3SS), alternative 5′ splice site (5SS), intron retention (IR), and microexon skipping (MIC). The annotation of U12-type introns used was as described in our previous paper [9], updating the U12 intron database [54] and generating position weight matrices for the donor, acceptor, and branch sites separately for U12-ATAC and u12-GTAG introns. Matrices were then used to scan the introns of interest and classify them as U2 or U12. Intron retention events corresponding to U12 events were determined using custom scripts, combining our results with the U12 intron database to check for U12 enrichment.

### 4.3. Reprogrammable Mice and iPSC Generation and Conversion

To generate reprogrammable mice combining mutant alleles of *Zrsr1* and *Zrsr2* and a ubiquitous doxycycline-inducible OSKM transgene that allowed us to produce iPSCs, we used the reprogrammable mouse line known as i4F-B [36], abbreviated as i4F. These i4F mice were crossed with *Zrsr1* and *Zrsr2* mutant mice. Heterozygotes for i4F, *Zrsr1*, and *Zrsr2* were crossed and fetuses at E13 were recovered to obtain mouse embryonic fibroblasts (MEFs) and produce iPSC. MEFs were prepared from mouse embryonic tissue as previously described [55]. For reprogramming, passage 1 fibroblasts were plated at a density of 5 × 10^5^ cells per well in six-well gelatin-coated plates and cultured in iPSC cell medium, namely high-glucose Dulbecco’s modified Eagle’s minimal essential medium supplemented with knockout serum replacement (15%, Invitrogen), leukemia inhibitory factor (1000 U/ mlL), nonessential amino acids, penicillin–streptomycin, glutamax, and β-mercaptoethanol with doxycycline (1 µg mL^−1^). The medium was changed every 48 h until iPSC-like colonies appeared (after ~7 days of treatment). Culture plates were stained for AP activity (AP detection kit, Chemicon International) on Day 7. The iPSC colonies were picked and maintained in feeder-free conditions using gelatin-coated plates (0.1% gelatin) and iPSC medium. The iPSCs were first transformed by electroporation with 2 µg of the linearized plasmid carrying an Emerald (a GFP variant) reporter driven by a 3.5 kb *Zscan4* promoter, which can reproduce the expression pattern of endogenous *Zscan4* in mouse ESCs [56]. Transformed cells were incubated for 10 days in growth medium supplemented with 10 µg/mL of Blasticidin S (Invivogen) until individual iPS cell clones were visible, then colonies were picked and propagated in iPSC medium. Then, the doxycycline-inducible mouse Dux-expression construct (TetO-3xHA-mDux) was used for a second transformation [27]. Stable cell lines were prepared by electroporation of 2 µg of linearized Dux plasmid. After recovery, cells were selected with puromycin (10 mg/mL) for 7 days before clones were picked and expanded. After their recovery, the cells were treated with doxycycline for 24 h to induce Dux transgene expression, as verified by RT-PCR.

### 4.4. Analysis of mRNA Levels by RT-qPCR

Murine preimplantation embryos were produced as previously reported [56]. Messenger RNA was extracted from 3 pools of 10 oocytes and 10 embryos at different developmental stages (zygote, 2-cell, 4-cell, morula, and blastocysts) using the Dynabeads mRNA Direct Extraction KIT (Dynal Biotech, Madrid, Spain) according to the manufacturer’s instructions. Immediately after extraction, the reverse transcription (RT) reaction was performed with the BioTaq enzyme (Bioline, London, UK) according to the manufacturer’s instructions. To prime the RT reaction and synthesize cDNA, poly(T) primer, random primers, and Moloney murine leukemia virus (MMLV) reverse transcriptase enzyme were used at a total volume of 40 µL. Tubes were heated to 70 °C for 5 min to denature the secondary RNA structure and the RT reaction was completed with the addition of 100 units of reverse transcriptase. The mixture was incubated at 42 °C for 60 min to allow the RT of RNA, which was followed by incubation at 70 °C for 10 min to denature the RT enzyme. Three groups of cDNA were set up for each experimental group with two replicates for all genes of interest. PCR was performed by adding a 2 µL aliquot of each sample to the PCR mix containing specific primers to amplify the genes of interest. Primer sequences are provided in Supplementary Table S7. Expression levels were normalized against that of the endogenous control *H2afz* as described previously [57]. The PCR conditions were optimized to achieve efficiencies close to 1. The comparative cycle threshold (CT) method was used to quantify expression levels. Fluorescence was acquired in each cycle to determine the threshold cycle or the cycle during the log-linear phase of the reaction at which fluorescence increased above the background level for each sample. Within this region of the amplification curve, a difference of one cycle is equivalent to doubling of the amplified PCR product. According to the comparative CT method, ΔCT was determined by subtracting the CT value obtained for the control gene (*H2afz*) from the CT value for each gene of interest in each sample. To calculate ΔΔCT, the highest sample ΔCT value (i.e., the sample showing the lowest target expression) was used as an arbitrary constant to subtract from all other ΔCT sample values. Fold changes in relative gene expression levels of target genes were determined using the formula 2^−ΔΔCT^. For cell culture qPCR, total RNA from at least three biological replicates was extracted using the TRIzol^®^ reagent (Invitrogen, CA, USA) and then treated with DNase (Promega, WI, USA) for 1 h. The concentration of RNA was determined by NanoDrop 2000 (ThermoScientific, MA, USA). DNase-treated total RNA (500 ng) was reverse-transcribed with oligo(dT) and SuperScript II (Invitrogen, CA, USA). RT, qPCR, and quantitative real-time PCR were performed as previously described. Gene expression levels were then normalized to those of *Gapdh* and *H2afz*.

### 4.5. Statistical Analysis

All data compiled from experiments were run in triplicate and reported as the mean ± SEM. All statistical tests were performed using the software package SigmaStat (Systat Software Inc., San Jose, CA, USA). Significant differences were determined based on the Student’s *t*-test (two groups), one-way ANOVA, or two-way ANOVA followed by Tukey’s post hoc test, unless otherwise stated. Significance was set at *p* < 0.05.

## Supplementary Materials

The following are available online at https://www.mdpi.com/1422-0067/21/11/4115/s1, Figure S1: Expression levels of U12-type introns containing genes and ZRSR1 and ZRSR2 during preimplantation embryo development. References [24,26,27,28] are cited in Supplementary Figure S1; Figure S2: Zrsr1 and Zrsr2 mutant mice lifespan, blockage of double mutant embryos and altered splicing events in Zrsr1/2mu 2-cell embryos; Table S1: Summary of differentially expressed genes, differentially expressed isoforms and altered splicing events in Zrsr1/2mu embryos; Table S2: Differentially expressed genes in Zrsr1/2mu and wild type (WT) 2-cell embryos; Table S3: Differentially expressed isoforms in Zrsr1/2mu and wild type (WT) 2-cell embryos; Table S4: Gene Ontology analysis of up- and down-regulated genes in Zrsr1/2mu 2-cell embryos; Table S5: List of alternative splicing events in Zrsr1/2mu and WT 2-cell embryos.
